# Unveiling the Potential of C_max_ *f*_2_ Factor Applied to Pilot Bioavailability/Bioequivalence Studies—A Case Study with Pazopanib Drug Products

**DOI:** 10.3390/pharmaceutics16121579

**Published:** 2024-12-11

**Authors:** Sara Carolina Henriques, Ana Leblanc, Sérgio Simões, Marlene Fonseca, Francisco Luís Pimentel, Luis Almeida, Nuno Elvas Silva

**Affiliations:** 1 BlueClinical Ltd., Senhora da Hora, 4460-439 Matosinhos, Portugal; aleblanc@blueclinical.pt (A.L.); mfonseca@blueclinical.pt (M.F.); fpimentel@blueclinical.pt (F.L.P.); lalmeida@blueclinical.pt (L.A.); 2 Research Institute for Medicines (iMed.ULisboa), Faculty of Pharmacy, Universidade de Lisboa, 1649-003 Lisbon, Portugal; 3 Bluepharma-Indústria Farmacêutica, S.A., São Martinho do Bispo, 3045-016 Coimbra, Portugal; ssimoes@ci.uc.pt; 4 Faculty of Pharmacy, University of Coimbra, Azinhaga de Santa Comba, 3000-548 Coimbra, Portugal; 5 Department of Biomedicine, Unit of Pharmacology & Therapeutics, Faculty of Medicine, University of Porto, 4200-450 Porto, Portugal; 6 MedInUP—Center for Drug Discovery and Innovative Medicines, University of Porto, 4200-450 Porto, Portugal

**Keywords:** bioequivalence, generic medicinal products, pilot studies, *f*_2_ factor, pharmacokinetics, highly variable drugs, pazopanib

## Abstract

**Background:** When companies are uncertain about the potential of a new formulation to be bioequivalent to a Reference product, it is common practice to carry out downsized pilot studies as a gatekeeping in vivo strategy to decide whether to move forward or not with a full-size pivotal study. However, due to the small study size, these studies are inarguably more sensitive to variability. **Objectives:** To address and mitigate the uncertainty of the conclusions of pilot studies concerning the maximum observed concentration (C_max_), the *f*_2_ factor was proposed as an alternative approach to the average bioequivalence statistical methodology. **Methods:** In this work, the alternative methodology is applied to pharmacokinetic data from pilot bioequivalence trials performed with pazopanib 200 mg and 400 mg. **Results:** Despite the small sample size, and very high intra-subject variability, the *f*_2_ factor demonstrated the potential for predicting bioequivalence. The positive results were confirmed in the full sized pivotal studies. **Conclusions:** In conclusion, this alternate methodology shows promise in reducing uncertainty associated with pilot studies and aiding in decisions to go forward with pivotal bioequivalence studies.

## 1. Introduction

Pilot bioavailability/bioequivalence (BA/BE) studies are downsized trials that can be conducted prior to pivotal trials. In these trials, 12 to 30 subjects are usually enrolled, although, in principle, a sample size is not formally calculated. Analysis and interpretation of results from pilot studies usually rely on the application of the average bioequivalence approach. However, due to the small study size, these studies are inarguably more sensitive to variability [1,2,3].

In order to overcome and reduce the uncertainty of the conclusions of pilot studies and on the potential of test formulations, Henriques et al. (2023) [1,2] proposed the *f*_2_ factor as an alternative approach to the average bioequivalence methodology to assess the potential bioequivalence for the maximum observed concentration (C_max_). C_max_ metric usually shows a higher variability in comparison to exposure metrics (area under the plasma concentration-time curve [AUC]), and therefore a demonstration of bioequivalence is commonly more difficult for C_max_. For the evaluation of pilot BA/BE studies, the authors suggest the application of average bioequivalence analysis, and additionally, the application of the *f*_2_ factor method in the case where the 90% confidence interval (CI) for geometric least square means ratio (GMR) is outside the [80.00–125.00]% regulatory acceptance bioequivalence interval. Using this alternative approach, and depending on *f*_2_ values and variability scenarios (20–60% IOV), the certainty levels to proceed with pivotal studies are highlighted. This is expected to assist companies in the decision-making process for proceeding with pivotal studies [1,2].

The proposed assessment was, however, solely based on simulated concentration-time profiles, and it is limited to drugs following a one compartment model, with median time of C_max_ (t_max_) ranging from 0.75 to 8 h, a short elimination half-life of (approximately 4.6 h), and a mean volume of distribution of approximately 60 L [1,2].

Hence, in order to validate the *f*_2_ factor method in a more diverse and realistic setting, the methodology was applied to real world pharmacokinetic data from BA/BE studies performed with pazopanib formulations.

Pazopanib is an ATP-competitive, second-generation inhibitor of tyrosine kinase activity associated with human vascular endothelial growth factor receptor (VEGFR)-1, VEGFR-2, VEGFR-3, platelet-derived growth factor receptor (PDGFR)-α and -β, fibroblast growth factor receptor (FGFR)-1 and -3, cytokine receptor (Kit), interleukin-2 receptor-inducible T-cell kinase (Itk), lymphocyte-specific protein tyrosine kinase (Lck), and transmembrane glycoprotein receptor tyrosine kinase (c-Fms). The drug is indicated for the treatment of patients with advanced renal cell carcinoma and for the treatment of patients with advanced soft tissue sarcoma who have received prior chemotherapy [4,5].

Pazopanib is a hydrochloride salt, being slightly soluble at pH 1 and insoluble above pH 4 in aqueous media. From in vitro caco-2 studies, pazopanib showed to be a highly permeable compound. Therefore, pazopanib is considered to be a class 2 compound (low solubility, high permeability) in the Biopharmaceutical Classification System (BCS).

Low pH dependent solubility is hypothesized to support an incomplete absorption of pazopanib, as well as a low and variable bioavailability (median was determined as 21% [range 14–39%]) [6].

From the literature, pazopanib is characterized by a low volume of distribution (approximately 25 L) [7,8]. Pharmacokinetic data from cancer patients were fitted to both monocompartment [7] or a two-compartment [8] population pharmacokinetic models, which highlights a non-extensive distribution into tissues. Following oral administration, pazopanib C_max_ is attained at approximately 2–8 h [6,9,10,11], which is within the *f*_2_ factor validation range. Moreover, pazopanib also shows high inter- and intra-subject variability for C_max_ and AUC [12,13], as tested in simulations for the validation of *f*_2_ factor [1,2].

However, pazopanib has a substantially longer elimination half-life, averaging approximately 30 h [6,9,10,11], deviating from the short half-life of approximately 4.6 h assumed in the simulated data [1,2]. Nevertheless, pazopanib pharmacokinetics was considered a suitable candidate to test the robustness of the *f*_2_ method in real-world pilot bioequivalence settings, as the main objective of this work. Results derived from *f*_2_ methodology were intended to assist the decision-making process in proceeding to pivotal studies.

## 2. Materials and Methods

Analyzes were performed on pharmacokinetic data obtained from two pilot and two pivotal BA/BE trials:

- Pazopanib 200 mg pilot study (BLCL-PAZ-PIL01, EudraCT No. 2020-00586-16):
  This study was a single-center, single-dose, open label, laboratory blinded, randomized, two-treatment, two-sequence, two-period (2 × 2 × 2) crossover pilot study in healthy male and nonpregnant female volunteers, with pazopanib 200 mg under fasting conditions. Doses of investigational product were separated by a washout of 14 days.
  A total of 24 healthy subjects received at least one dose of investigational product and 23 subjects completed this study and were included in the statistical analysis.
- Pazopanib 200 mg pivotal study (BLCL-PAZ-EU-02, EudraCT No. 2021-002053-29):
  This study was a single-center, single-dose, open label, laboratory blinded, randomized, two-treatment, two-sequence, two-period (2 × 2 × 2) crossover pivotal study in healthy male and nonpregnant female volunteers, with pazopanib 200 mg under fasting conditions. Doses of investigational product were separated by a washout of 21 days.
  In total, 116 healthy subjects received at least one dose of investigational product. From these, 106 subjects completed this study and were included in the statistical analysis.
- Pazopanib 400 mg pilot study (BLCL-PAZ-PIL02, EudraCT No. 2020-00514-17):
  This study was a single-center, single-dose, open label, laboratory blinded, randomized, two-treatment, two-sequence, two-period (2 × 2 × 2) crossover pilot study in healthy male and nonpregnant female volunteers, with pazopanib 400 mg under fasting conditions. Doses of investigational product were separated by a washout of 21 days.
  A total of 24 healthy subjects received at least one dose of investigational product and 23 subjects completed this study and were included in the statistical analysis.
- Pazopanib 400 mg pivotal study (BLCL-PAZ-EU-03, EudraCT No. 2021-003534-36):
  This study was a single-center, single-dose, open label, laboratory blinded, randomized, two-treatment, two-sequence, two-period (2 × 2 × 2) crossover pivotal study in healthy male and nonpregnant female volunteers, with pazopanib 400 mg under fasting conditions. Doses of investigational product were separated by a washout of 21 days.
  In total, 122 healthy subjects received at least one dose of investigational product. From these, 98 subjects completed this study and were included in the statistical analysis.

The current pivotal BA/BE trials were performed to support the marketing authorization application (MAH) of a generic pazopanib formulation (Test product), using Votrient^®^ as the Reference product. According to the European Medicines Agency (EMA)’s Guideline on the Investigation of Bioequivalence [14], for drugs with a less than proportional increase in area under the concentration-time curve (AUC) with an increasing dose over the therapeutic dose range, which is the case for pazopanib [15], bioequivalence should be established both at the highest strength and at the lowest strength. Hence, two single dose pivotal BA/BE studies under fasting condition were performed, with 200 mg and 400 mg to support MAH within EMA ambiance [15].

Before conducting the pivotal studies, the company performed two pilot studies under fasting conditions to obtain exploratory information on the relative bioavailability of the two developed formulations of pazopanib (200 mg and a 400 mg) in comparison to the Reference products, and to assist on the decision to go forward with pivotal studies.

Both pilot studies showed a Test-to-Reference GMR within the [80.00–125.00]% comparable bioavailability acceptance limits for C_max_; however, the corresponding 90% CI were outside the acceptance limits, hence, failing to show bioequivalence. The a posteriori pivotal studies, performed with an increased number of subjects, showed bioequivalence between the Test and Reference formulations.

The pharmacokinetic analysis was performed in the parent drug, as specified in the EMA’s pazopanib product-specific bioequivalence guidance [15]. Pazopanib plasma concentrations were measured using a validated liquid chromatography with tandem mass spectrometry (LC-MS/MS) analytical method in compliance with Good Laboratory Practices (GLP). The analytical method used a calibration range of 50 to 30,000 ng/mL, for the 200 mg dose, and a range of 100 to 60,000 ng/mL, for the 400 mg dose.

For each study, a total of 20 venous blood samples (volume of 6 mL each) per study period were scheduled for the quantification of pazopanib in plasma, at the following timepoints: pre-dose (t = 0 h); 0.50, 1.00, 1.50, 2.00, 2.50, 2.75, 3.00, 3.25, 3.50, 4.00, 4.50, 5.00, 6.00, 8.00, 10.00, 12.00, 24.00, 48.00, and 72.00 h post-dose.

The four studies were conducted at BlueClinical Phase I, Hospital da Prelada, Porto, Portugal.

Pharmacokinetic and average bioequivalence analyzes were performed with Phoenix^®^ WinNonlin^®^ 8.2 (Certara USA Inc., Princeton, NJ, USA). Additional analyzes and graphics were performed with R version 4.3.2 (R Foundation for Scientific Computing, Vienna, Austria, 2013).

As defined by the EMA’s Guideline on the Investigation of Bioequivalence [14], only subjects who provided evaluable pharmacokinetic data for both test and reference products were used for statistical analysis. A summary of the main demographic characteristics of the subjects included in the statistical analysis, for each study, is presented in Table 1.

### 2.1. Average Bioequivalence Analysis

Pharmacokinetic metrics C_max_, time of occurrence of C_max_ (t_max_), AUC from time of dosing (t = 0 h) truncated at 72 h (AUC_0–72_), apparent terminal elimination rate constant (λ_z_), and apparent terminal elimination half-life (t_1/2_) were estimated from individual pharmacokinetic profiles for each formulation, by using a non-compartmental analysis (NCA) approach with a *ln*-linear terminal phase assumption.

For each study, the assessment of the similarity on the rate of drug absorption, following the administration of Test and Reference products, was performed using an analysis of variance (ANOVA) applied to the *ln*-transformed C_max_ and estimating the Test-to-Reference least square-means (LSM) ratio (GMR) and the corresponding 90% confidence interval (CI). The ANOVA model included Sequence, Subject nested within Sequence, and Period and Formulation as fixed effects, assessed at a 5% significance level (α = 0.05) [14]. The intra-subject coefficient of variation (ISCV) was estimated from the mean square error (*s*^2^) of the ANOVA model, as:

(1) $$ISCV=100\cdot \sqrt{e^{s^{2}}-1}$$

As per EMA’s bioequivalence guideline, bioequivalent between the Test and Reference products is demonstrated if the GMR and corresponding 90% CI fell within the [80.00–125.00]% acceptance interval [14].

### 2.2. Alternative f_2_ Factor Approach

As an alternative to the average bioequivalence approach, the *f*_2_ factor was used to assess the similarity on the rate of drug absorption as proposed by Henriques et al. (2023) [1,2], using the R package ‘f2PilotBE’ [16].

The *f*_2_ factor was calculated by normalizing the mean Test and Reference concentration-time profiles to the C_max_ of the mean Reference profile, until C_max_ of the mean Reference profile was observed (Reference t_max_) [1,2]:

(2) $$C_{t}^{N}=100\cdot \frac{{\bar{C}}_{t}}{{C_{max}}_{R}}\;\;\;where\;0\leq t\leq {t_{max}}_{R},$$

(3) $${C_{max}f}_{2}=50\cdot log\left({100\cdot \left[1+\frac{1}{n}\sum_{t=1}^{t=n}{\left(R_{t}^{N}-T_{t}^{N}\right)}^{2}\right]}^{-0.5}\right)$$

A cut-off of 35 was considered to indicate a similarity between the Test and Reference products C_max_ (assessing the rate of absorption) [1,2]. Considering that pazopanib is a highly variable drug, the following conclusions can be taken based on the magnitude of the estimated *f*_2_ factor [2]:

- If *f*_2_ factor ≥ 35 and ISCV > 40%, the confidence in proceeding to a pivotal study is >60%.
- If *f*_2_ factor ≥ 41 and ISCV ≥ 50%, the confidence in proceeding to a pivotal study is >80%.
- If *f*_2_ factor ≥ 50, the confidence in proceeding to a pivotal study is high (>90%) regardless of ISCV.

## 3. Results

### 3.1. Pazopanib 200 mg

**Figure 1:**
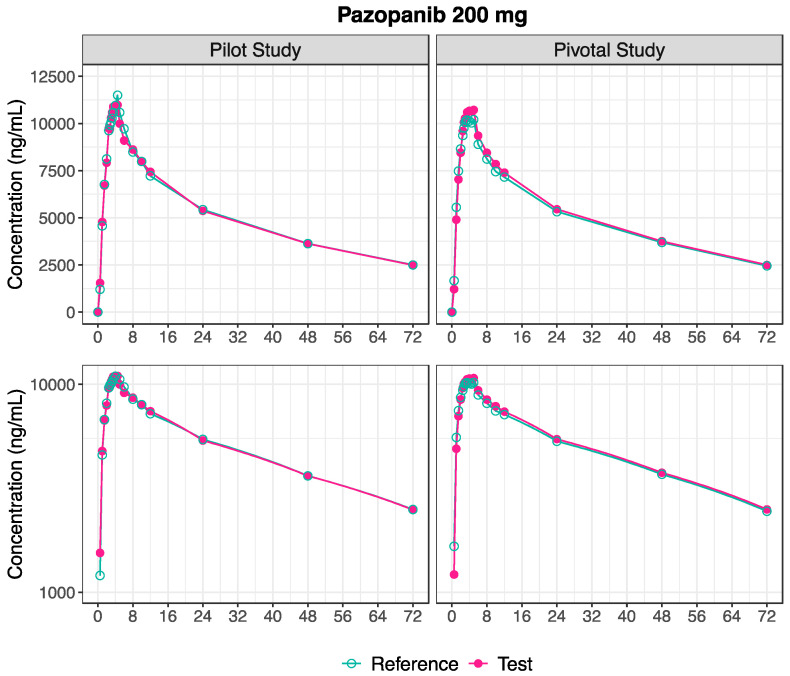
Plasma concentration-time profiles following the administration of pazopanib 200 mg Test and Reference formulation, in pilot (n = 23, **Left**) and pivotal (n = 106, **Right**) studies, in linear (**Above**) and semi-logarithmic (**Below**) scales.

**Figure 2:**
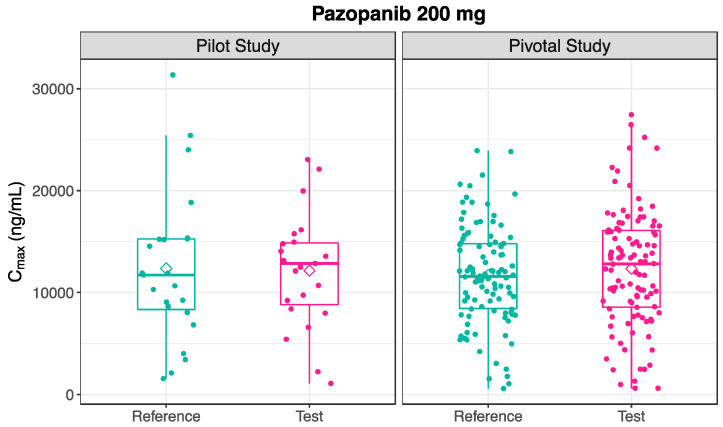
Box plots of distribution of C_max_ following the administration of pazopanib 200 mg Test and Reference formulation, in pilot (n = 23, **Left**) and pivotal (n = 106, **Right**) studies. Individual values are represented as points, while median and mean values are represented as a line and a diamond shape, respectively.

Following the administration of Test and Reference products in the pilot study, the mean pharmacokinetic profiles are illustrated in Figure 1, and the estimated pharmacokinetic metrics are presented in Table 2. The distribution of the estimated C_max_ after the 200 mg administration of the Test and Reference formulation of pazopanib is visually depicted in Figure 2.

**Figure 3:**
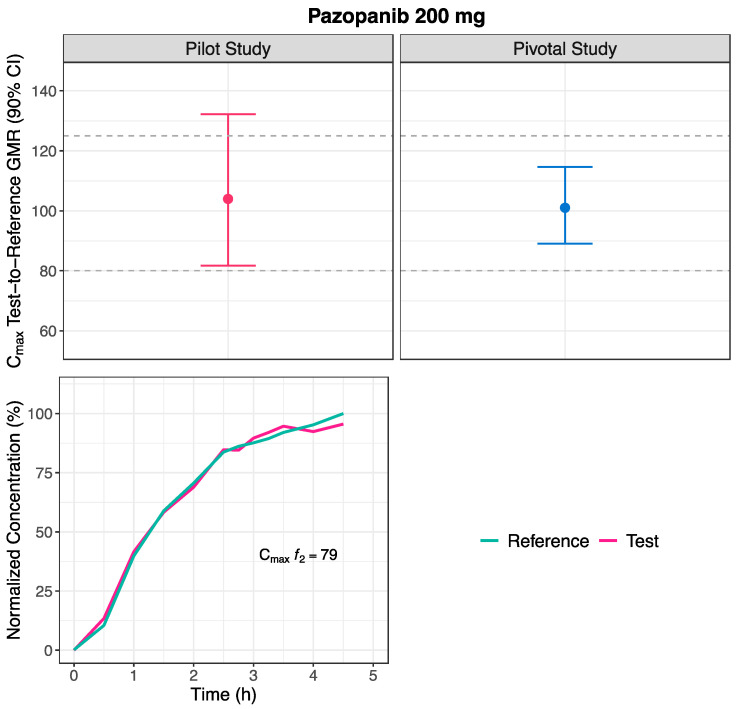
Test-to-Reference geometric least square means ratio (GMR) and corresponding 90% confidence interval (CI) estimated for C_max_ (**Above**), and normalized Test and Reference mean concentration-time curves, to the Reference C_max_, until the Reference t_max_, for the estimation of the similarity *f*_2_ factor (**Below**), following the administration of pazopanib 200 mg, in pilot (n = 23, **Left**) and pivotal (n = 106, **Right**) studies.

The point estimate calculated from the average bioequivalence for C_max_ was close to 100%, and within the [80.00–125.00]% comparable bioavailability acceptance limits. Likewise, the lower limit of the GMR 90% CI fell within the [80.00–125.00]% comparable bioavailability acceptance limits. However, the upper limit of the GMR 90% CI was above the [80.00–125.00]% comparable bioavailability acceptance limits (Figure 3 and Table 3). Moreover, the estimated ISCV was high (50.1%) (Table 3).

Using the alternative *f*_2_ factor approach, the mean Test and Reference concentration-time profiles (Figure 1) were normalized to the C_max_ of the Reference mean profile. The normalization was performed until 4.5 h for the pilot study (Figure 3). The calculated *f*_2_ factor from the normalized profiles was 79.00 (Figure 3 and Table 3).

Following the administration of the Test and Reference products in the pivotal study, the mean pharmacokinetic profiles are illustrated in Figure 1 and the estimated pharmacokinetic metrics are presented in Table 2. The pharmacokinetic outcomes for the 200 mg pazopanib were consistent across both pilot and pivotal studies. Figure 2 shows a similar distribution of C_max_ values following the administration of pazopanib 200 mg Test and Reference formulations between the pilot and pivotal studies.

For the pivotal study, the estimated GMR for C_max_ was close to 100% and the corresponding 90% CI fell within the [80.00–125.00]% comparable bioavailability acceptance limits (Figure 3 and Table 3). Likewise, the estimated ISCV was high (59.8%) (Table 3).

### 3.2. Pazopanib 400 mg

**Figure 4:**
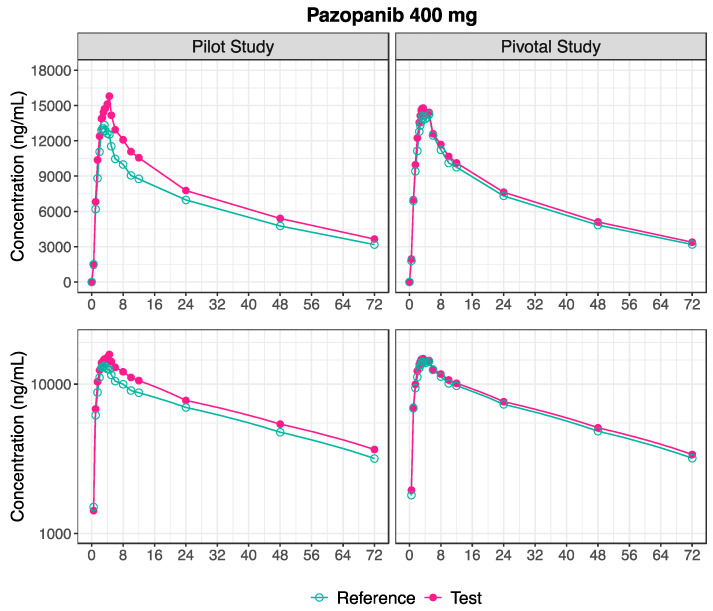
Plasma concentration-time profiles following the administration of pazopanib 400 mg Test and Reference formulation, in pilot (n = 23, **Left**) and pivotal (n = 98, **Right**) studies, in linear (**Above**) and semi-logarithmic (**Below**) scales.

**Figure 5:**
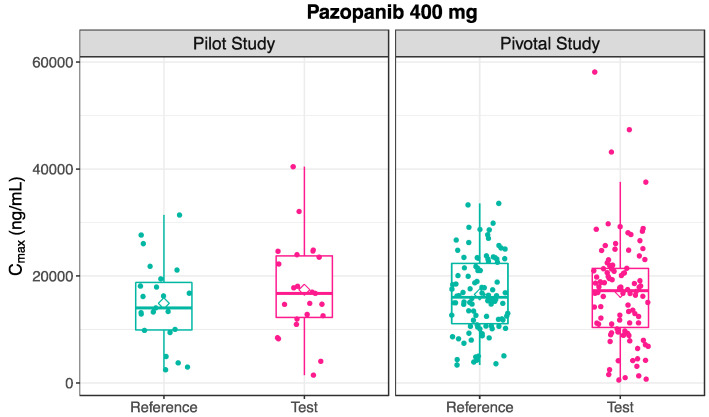
Box Plots of Distribution of C_max_ Following the Administration of Pazopanib 400 mg Test and Reference Formulation, in Pilot (n = 23, **Left**) and Pivotal (n = 98, **Right**) Studies. Individual Values are Represented as Points, While Median and Mean Values are Represented as a Line and a Diamond Shape, respectively.

Following the oral administration of 400 mg pazopanib in the pilot study, the mean plasma concentration-time profile for the Test product was slightly higher than the Reference mean profile (Figure 4). The estimated pharmacokinetic metrics are presented in Table 4, and the distribution of the estimated C_max_ after the 400 mg administration of the Test and Reference formulation of pazopanib is visually depicted in Figure 5.

**Figure 6:**
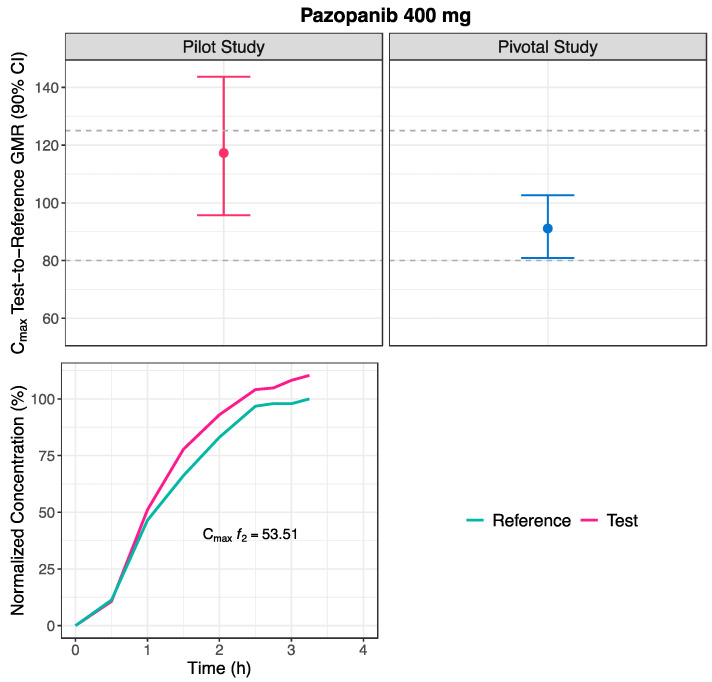
Test-to-Reference geometric least square means ratio (GMR) and corresponding 90% confidence interval (CI) estimated for C_max_ (**Above**), and normalized Test and Reference mean concentration-time curves, to the Reference C_max_, until the Reference t_max_, for the estimation of the similarity *f*_2_ factor (**Below**), following the administration of pazopanib 400 mg, in the pilot (n = 23, **Left**) and pivotal (n = 98, **Right**) studies.

In the pilot study, the point estimate calculated from the average bioequivalence for C_max_ was above 100%, but within the [80.00–125.00]% comparable bioavailability acceptance limits. Likewise, the lower limit of the GMR 90% CI fell within the [80.00–125.00]% comparable bioavailability acceptance limits. Nevertheless, the upper limit of the GMR 90% CI was above the [80.00–125.00]% comparable bioavailability acceptance limits (Figure 6 and Table 5). Moreover, the estimated ISCV was high (41.7%) (Table 5).

Using the alternative *f*_2_ factor approach, the mean Test and Reference concentration-time profiles (Figure 4) underwent normalization to the C_max_ of the Reference mean profile. The normalization was carried out until 3.25 h (Figure 6). The calculated *f*_2_ factor from the normalized profiles was 53.51 (Figure 6 and Table 5).

However, the pharmacokinetic outcomes for the 400 mg pazopanib were slightly different between the pilot and pivotal studies. With the increment of subjects in the pivotal study (n = 98), the mean plasma concentration-time profiles following the administration of the Test and Reference products were almost superimposable. These profiles are visually depicted in Figure 4. The estimated pharmacokinetic metrics are presented in Table 4, and Figure 5 shows a similar distribution of C_max_ values following the administration of the Test and Reference formulations of 400 mg pazopanib.

The increment in the number of subjects to 98 allowed the GMR and the corresponding 90% CI to fall within the [80.00–125.00]% comparable bioavailability acceptance limits. Nevertheless, in opposition to the pilot study, in the pivotal study, the GMR decreased to below 100% (Figure 6 and Table 5). Likewise, the estimated ISCV was high (53.2%) (Table 5).

## 4. Discussion

Pazopanib is recognized as a highly variable drug [12,13], which was also observed in the current pivotal bioequivalence studies, with an ISCV of approximately 50% for both C_max_ and AUC bioequivalence metrics. Such high variability is probably due to the variable absorption as a consequence of a variable and low pH dependent solubility of the compound on the form of hydrochloride salt [6].

### 4.1. Pazopanib 200 mg

In the pilot study performed with 200 mg pazopanib formulations, bioequivalence between the Test and Reference products was not demonstrated for the 23 subjects included in the statistical analysis. However, the point estimate was centered, indicating proximity to 100%. Nevertheless, it is important to note that, based on prior simulations, a centered GMR alone suggests only a ~60% probability of the Test product being truly bioequivalent, particularly in scenarios with high ISCV [1,2].

Upon applying the alternative approach, the estimated *f*_2_ factor was 79.00, which, in accordance with conclusions drawn from previous simulations, i.e., for an *f*_2_ factor of this dimension (>50), indicates a 90% probability that the Test product is truly bioequivalent to the Reference product in terms of C_max_. Therefore, the sponsor could be recommended to proceed with a full-size pivotal study [2].

Considering the results from the pilot study, sponsor conducted a pivotal study with 116 subjects. A sample of 96 subjects would allow an a priori statistical power of at least 80% to meet the [80.00–125.00]% bioequivalence range, assuming an ISCV of 50% for AUC metric, a true GMR of 105%, and a 5% significance level (α = 0.05). To compensate for possible dropouts and variation in ISCV, a sample size of 116 subjects was defined. From the 116 enrolled subjects, 106 completed this study and had evaluable pharmacokinetic data for bioequivalence analysis. The increment of the number of subjects to 106 allowed the GMR and the corresponding 90% CI to fall completely within the [80.00–125.00]% acceptance interval; hence, showing bioequivalence.

The values derived for C_max_, t_max_, and t_1/2_ for both pilot and pivotal studies are in agreement with results from a bioequivalence study performed in healthy Chinese subjects [13]. However, the AUC parameter is not comparable due to different last sampling times.

### 4.2. Pazopanib 400 mg

Similarly to the 200 mg pazopanib, in the pilot study performed with 400 mg pazopanib formulations, bioequivalence between the Test and Reference products was not demonstrated for the 23 subjects included in the statistical analysis. Hence, the point estimate was above 100% and not centered, but within the [80.00–125.00]% comparable bioavailability acceptance limits, along with its lower limit of the 90% CI.

Upon applying the alternative approach, the estimated *f*_2_ factor was 53.51. According with conclusions from previous simulations, an *f*_2_ factor of this dimension (>50) indicates a 90% probability that the Test product is truly bioequivalent to the Reference product in terms of C_max_. Therefore, the sponsor could be recommended to proceed with a full-size pivotal study [2].

The sponsor decided to conduct a pivotal study with 122 subjects. A sample of 106 subjects would allow an a priori statistical power of at least 80% to meet the [80.00–125.00]% bioequivalence range, assuming an ISCV of 35%, a true GMR falling within [90.00–111.11]%, and a 5% significance level (α = 0.05). To compensate for possible dropouts and variation in ISCV, a sample size of 122 subjects was defined. From the 122 enrolled subjects, 98 completed this study and had evaluable pharmacokinetic data for bioequivalence analysis. The increase in the number of subjects to 98 allowed the GMR and the corresponding 90% CI to fall completely within the [80.00–125.00]% acceptance interval; hence, showing bioequivalence.

To date, no bioequivalence studies have been published with 400 mg strength. Nevertheless, the elimination half-life observed is similar to values found for the 200 mg strength in the performed pivotal study and the literature [13].

Moreover, when comparing results derived from the 400 mg and 200 strengths, a less than dose proportional behavior in C_max_ and AUC_0-72_ was observed, as described [6,9], primarily driven by limited solubility and saturation of absorption processes at higher doses.

## 5. Conclusions

The *f*_2_ factor has shown to be capable of predicting bioequivalence between two pazopanib formulations for both 200 mg and 400 mg strengths, using data from pilot studies, despite the limited sample size of only 23 subjects that have been involved, considered to be low facing the high intra-subject variability known for the drug.

The methodology is intended to be applied to data from other bioequivalence trials covering BCS class 2 and class 4 drugs. Nevertheless, the *f*_2_ factor has been exhibiting the potential to reduce the uncertainty associated with pilot studies and to be helpful in terms of making the decision to go forward with pivotal bioequivalence studies.

## Figures and Tables

**Table 1:** Summary of demographic characteristics of subject included in the statistical analysis for each study.

Demography	Pazopanib 200 mg		Pazopanib 400 mg	
	Pilot Study (n = 23)	Pivotal Study (n = 106)	Pilot Study (n = 23)	Pivotal Study (n = 98)
Sex				
Male	6 (26.1%)	48 (45.3%)	14 (60.9%)	43 (43.9%)
Female	17 (73.9%)	58 (54.7%)	9 (39.1%)	55 (56.1%)
Race				
Black or African American	1 (4.3%)	7 (6.6%)	0 (0.0%)	6 (6.1%)
Multiple	5 (21.7%)	12 (11.3%)	2 (8.7%)	19 (19.4%)
White	17 (73.9%)	87 (82.1%)	21 (91.3%)	73 (74.5%)
Age (years)	30 (22–42)	29 (18–48)	29 (19–50)	31 (18–51)
Height (cm)	168 (144–193)	168 (152–190)	172 (157–190)	168 (150–191)
Weight (kg)	69.0 (47.6–106.2)	68.3 (47.7–94.2)	69.1 (52.0–97.8)	69.8 (45.2–94.0)
BMI (kg/m^2^)	24.3 (18.8–29.6)	24.2 (18.6–29.9)	23.2 (18.9–28.9)	24.6 (15.5–30.0)

n—Number of subjects with information for both the Test and Reference formulations; BMI—Body mass index. Categorical data are summarized as n, and the percentage is within the parenthesis. Continuous data are summarized as mean with the range within the parenthesis.

**Table 2:** Summary statistics of pharmacokinetic metrics following the administration of pazopanib 200 mg, in pilot (n = 23) and pivotal (n = 106) studies.

	Pazopanib 200 mg			
	Pilot Study		Pivotal Study	
Metric (Unit)	Test (n = 23)	Reference (n = 23)	Test (n = 106)	Reference (n = 106)
C_max_ (ng/mL)	12,153.87 (45.6%)	12,377.02 (59.9%)	12,342.44 (46.5%)	11,713.78 (41.2%)
t_max_ (h)	3.25 (1.50–8.00)	4.00 (1.50–6.00)	3.25 (1.00–10.00)	3.13 (1.00–10.00)
AUC_0–72_ (ng·h/mL)	354,866.48 (44.6%)	354,966.79 (51.2%)	359,463.36 (44.9%)	350,094.25 (43.0%)
λ_z_ (1/h)	0.017 (30.3%)	0.018 (28.6%)	0.017 (21.3%)	0.017 (21.6%)
t_1/2_ (h)	44.91 (28.4%)	42.69 (30.5%)	42.84 (22.5%)	42.17 (23.7%)

C_max_—Maximum observed concentration; t_max_—Time to maximum observed concentration; AUC_0–72_—Area under the concentration-time curve (AUC) truncated at 72 h (AUC from time of dosing up to 72 h); λ_z_—apparent terminal elimination rate constant; t_1/2_—apparent terminal elimination half-life. Values are the arithmetic mean with the coefficient of variation (CV%) within the parenthesis. For t_max_, values are the median with the range between the parentheses.

**Table 3:** Average Bioequivalence and *f*_2_ Factor Analysis Results for C_max_ Following the Administration of Pazopanib 200 mg.

	Pazopanib 200 mg	
	Pilot Study	Pivotal Study
n	23	106
ISCV (%)	50.1	59.8
Geometric LSM (ng/mL)		
Test	10,398.64	10,427.44
Reference	10,001.77	10,322.74
GMR [90% CI] (%)	103.97 [81.76–132.21]	101.01 [89.04–114.60]
*f*_2_ Factor	79.00	NC

n—Number of subjects with information for both the Test and Reference formulations; ISCV—Intra-subject coefficient of variation (calculated from the average bioequivalence); LSM—Least square mean (calculated from the average bioequivalence); GMR—Test-to-Reference geometric coefficient of variation (calculated from the average bioequivalence); CI—Confidence interval of the GMR (calculated from the average bioequivalence); NC—Not calculated.

**Table 4:** Summary Statistics of Pharmacokinetic Metrics Following the Administration of Pazopanib 400 mg, in Pilot (n = 23) and Pivotal (n = 98) Studies.

	Pazopanib 400 mg			
	Pilot Study		Pivotal Study	
Metric (Unit)	Test (n = 23)	Reference (n = 23)	Test (n = 98)	Reference (n = 98)
C_max_ (ng/mL)	17,413.79 (51.1%)	14,894.93 (51.7%)	17,031.50 (57.4%)	16,552.74 (42.8%)
t_max_ (h)	3.00 (1.50–4.50)	2.75 (1.00–4.50)	3.50 (1.00–10.00)	3.25 (1.00–6.00)
AUC_0–72_ (ng·h/mL)	512,126.67 (57.3%)	444,128.55 (58.0%)	492,949.12 (59.0%)	469,368.48 (46.5%)
λ_z_ (1/h)	0.018 (21.3%)	0.018 (23.9%)	0.018 (23.0%)	0.018 (25.6%)
t_1/2_ (h)	41.56 (21.1%)	41.02 (24.7%)	41.49 (35.0%)	40.86 (25.4%)

C_max_–Maximum observed concentration; t_max_—Time to maximum observed concentration; AUC_0-72_—Area under the concentration-time curve (AUC) truncated at 72 h (AUC from time of dosing up to 72 h); λ_z_—apparent terminal elimination rate constant; t_1/2_—apparent terminal elimination half-life. Values are the arithmetic mean with the coefficient of variation (CV%) within the parenthesis. For t_max_, values are the median with the range between the parentheses.

**Table 5:** Average Bioequivalence and *f*_2_ Factor Analysis Results for C_max_ Following the Administration of Pazopanib 400 mg.

	Pazopanib 400 mg	
	Pilot Study	Pivotal Study
n	23	98
ISCV (%)	41.7	53.2
Geometric LSM (ng/mL)		
Test	14,667.79	13,429.92
Reference	12,509.20	14,739.56
GMR [90% CI] (%)	117.26 [95.69–143.68]	91.11 [80.91–102.61]
*f*_2_ Factor	53.51	NC

n—Number of subjects with information for both the Test and Reference formulations; ISCV—Intra-subject coefficient of variation (calculated from the average bioequivalence); LSM—Least square mean (calculated from the average bioequivalence); GMR—Test-to-Reference geometric coefficient of variation (calculated from the average bioequivalence); CI—Confidence interval of the GMR (calculated from the average bioequivalence); NC—Not calculated.

## Data Availability

The data that support the findings of this study are available on request to the corresponding authors. The data are not publicly available due to privacy or ethical restrictions.
